# Amoxapine-induced tardive dyskinesia

**DOI:** 10.4103/0019-5545.58305

**Published:** 2009

**Authors:** Gurvinder Pal Singh

**Affiliations:** Department of Psychiatry, Government Medical College and Hospital, H.No.1007/1, Sector 46-B, Chandigarh - 160 046, India. E-mail: gpsluthra@rediffmail.com

Sir,

Modern clinical practice presents psychiatrists with a choice of numerous medications to treat various ailments. Persistent tardive dyskinesia associated with amoxapine therapy has been reported previously in international literature two decades ago.[1–5] To the best of the author's knowledge, no prior similar case report, focusing on this clinical problem, has been reported from north India. Thus, a strong need was felt to report this case.

Mr. VK, a 49-year-old patient, presented to our Psychiatric Center in November 2004, with a psychiatric illness. The illness was of a six-month duration, gradual in onset and without any precipitating factors. He had complaints of sadness of mood, suspiciousness, delusions of persecution, and depressive ideas. He remained predominantly anxious and had decreased sleep and constipation. He also complained of difficulty in performing sexual intercourse with his wife due to poor erection. The patient had associated diabetes mellitus and was on oral hypoglycemic agents from a private physician. On mental status examination, the patient was anxious, with slow speech, and had depressive thoughts. All his routine and fasting blood sugar levels were within normal range. The patient had a past history of similar complaints four years ago and showed marked improvement with amoxapine, and remained under treatment for two years and then the medication was stopped.

On account of a good response with this medication in the past, the patient was put on amoxapine 100 mg/day and clonazepam 0.5 mg/day. After two weeks of medication he came with his wife for a follow-up and reported improvement. The doses of clonazepam were reduced to half of the initial dose. With this treatment his anxiety symptoms lessened in severity, and he seemed to be in a better mood most of the time. However, the patient still had suspicious thoughts related to his medication. After one month of treatment the dose of amoxapine was increased to 150 mg.

The patient came regularly for follow-up for two years and was much better with medications. His wife reported that since November 2006, when the patient was sitting alone, he had irregular movements of his hand and lips. On detailed examination, it was observed that the movements of the patient were involuntary and involved the tongue, lips, and the right and left hands. The movements were stereotyped (patterned and repetitive). Perioral movements (tongue twisting/protrusion, facial grimacing, lip puckering) and hand clenching and finger movements were also present in this patient. All his psychotropic medications were discontinued and the patient was treated with escitalopram (10 mg/day) and tetrabenazine (50 mg/ daily) and was stabilized on this dose over the next four weeks. With combined therapy he showed significant improvement and his irregular movements disappeared. His depressive thoughts diminished in intensity and he started participating in social activities.

Amoxapine is a tricyclic antidepressant of the dibenzoxazepine class. The mechanism of the clinical action of amoxapine in man is not well understood. Its major metabolite 7-hydroxyamoxapine has a dopamine receptor blocking effect. Unfortunately, tardive dyskinesia is a secondary focus of research in the current clinical practice among patients on antidepressants. The risk of a patient developing tardive dyskinesia, and of the syndrome becoming irreversible, appears to increase with the duration of treatment and the total amount of drugs administered. This problem is relatively under-recognized in the clinical setting.[6] Data suggest that patients suffering from mood disorders are at a higher risk of developing tardive dyskinesia, compared to patients with schizophrenia. This report warns clinicians that amoxapine may not be an ideal choice of antidepressant, even for psychotic depression.
